# Nitrogen effects on the pelagic food web are modified by dissolved organic carbon

**DOI:** 10.1007/s00442-017-3921-5

**Published:** 2017-07-29

**Authors:** A. Deininger, C. L. Faithfull, A.-K. Bergström

**Affiliations:** Department of Ecology and Environmental Science, Umeå University, Umeå, Sweden

**Keywords:** Boreal lakes, Global change, Nitrogen availability, Trophic transfer efficiency, Zooplankton

## Abstract

Global environmental change has altered the nitrogen (N) cycle and enhanced terrestrial dissolved organic carbon (DOC) loadings to northern boreal lakes. However, it is still unclear how enhanced N availability affects pelagic food web efficiency (FWE) and crustacean zooplankton growth in N limited boreal lakes. Here, we performed in situ mesocosm experiments in six unproductive boreal Swedish lakes, paired across a DOC gradient, with one lake in each pair fertilized with N (2011: reference year; 2012, 2013: impact years). We assessed how zooplankton growth and FWE were affected by changes in pelagic energy mobilization (PEM), food chain length (phytoplankton versus bacterial production based food chain, i.e. PP:BP), and food quality (seston stoichiometry) in response to N fertilization. Although PP, PEM and PP:BP increased in low and medium DOC lakes after N fertilization, consumer growth and FWE were reduced, especially at low DOC—potentially due to reduced phytoplankton food quality [increased C: phosphorus (P); N:P]. At high DOC, N fertilization caused modest increases in PP and PEM, with marginal changes in PP:BP and phytoplankton food quality, which, combined, led to a slight increase in zooplankton growth and FWE. Consequently, at low DOC (<12 mg L^−1^), increased N availability lowers FWE due to mismatches in food quality demand and supply, whereas at high DOC this mismatch does not occur, and zooplankton production and FWE may increase. We conclude that the lake DOC level is critical for predicting the effects of enhanced inorganic N availability on pelagic productivity in boreal lakes.

## Introduction

Human activities (e.g. land use changes, usage of fertilizers, and burning of fossil fuels) have changed the global nitrogen (N) cycle (Vitousek et al. 1997; Rockström et al. 2009; Greaver et al. 2016) and contributed to enhanced availability of inorganic N in northern boreal lakes (Bergström et al. 2005; Elser et al. 2009b). Many of these lake ecosystems are currently experiencing increased input of terrestrial dissolved organic carbon (DOC) mediated by warming, increased precipitation, and reduced atmospheric sulfate deposition (Monteith et al. 2007; de Wit et al. 2016; Finstad et al. 2016). Atmospheric N deposition caused by the anthropogenic release of inorganic nitrogen (N) has enhanced lake water N to phosphorus (P) stoichiometry, shifted nutrient limitation in phytoplankton from N to P limitation (Elser et al. 2009a; Hessen 2013), and enhanced phytoplankton biomass (Jansson et al. 2001; Deininger et al. 2017a). Despite this knowledge, we know little about how efficiently any additional phytoplankton primary production (PP) following increased N availability is transferred to crustacean zooplankton, and whether differences in lake DOC concentration will affect zooplankton responses. Since zooplankton provide an important link between basal producers and commercially and recreationally important fish species, it is crucial to address how energy will be transferred via zooplankton in order to fully understand how enhanced N availability affects the functioning of boreal lake ecosystems.

**Fig. 1:**
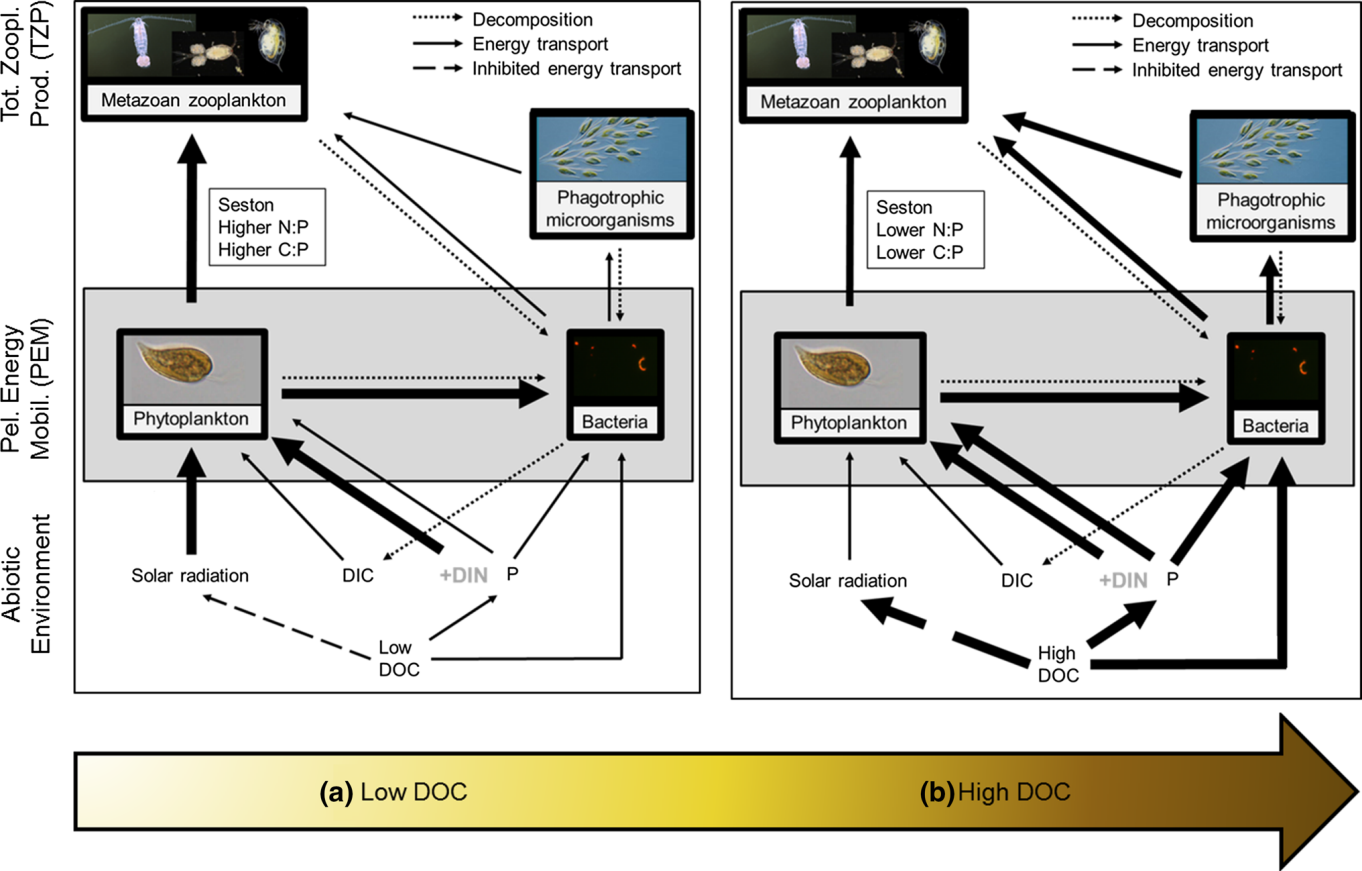
Conceptual model illustrating the predicted response of the boreal pelagic food chain to increased dissolved inorganic nitrogen (DIN) availability in lakes with (**a**) low dissolved organic carbon (DOC) and (**b**) high DOC. *Thickness* of *arrows* represents pathway strength. Other abbreviations are total pelagic energy mobilization (PEM), total zooplankton production (TZP), dissolved inorganic carbon (DIC), and phosphorus (P)

At present, phytoplankton in northern boreal lakes that have been exposed to low atmospheric N deposition are primarily N limited (Bergström et al. 2005; Elser et al. 2009b; Deininger et al. 2017b). Thus, an increased N input to these lake ecosystems should enhance phytoplankton production and biomass (Fig. 1). However, as photosynthesis is light dependent, a simultaneous increase in terrestrial lake DOC and an associated decrease in light availability (Jones 1992) should weaken, or even offset increased phytoplankton productivity caused by elevated N availability (Seekell et al. 2015; Deininger et al. 2017a). On the other hand, changes in terrestrial DOC input may also stimulate PP through enhanced nutrient availability, since DOC is a structural component of terrestrial organic matter which serves as an important carrier of N and phosphorus (P) to boreal lakes (Jansson et al. 2001; Jones et al. 2012). Importantly, any change in phytoplankton abundance following enhanced inorganic N availability is likely to directly affect consumers, since growth of crustacean zooplankton consumers in unproductive boreal lakes often is limited by food availability, i.e. phytoplankton (Persson et al. 2007).

However, crustacean zooplankton can obtain energy mobilized through both phytoplankton and bacterioplankton pathways (Jones 1992; Jansson et al. 2007). The sum of PP and bacterial production (BP) can, therefore, be used to classify the total pelagic energy mobilization (PEM) and the amount of energy available to crustacean consumers in the pelagic zone (Berglund et al. 2007). The PEM has been shown to be similar in N limited boreal clear and humic lakes; however, with increasing DOC, the contribution of BP to PEM increases, whereas the contribution of PP to PEM decreases (Faithfull et al. 2015). Thus, basal energy can reach zooplankton consumers via two pathways depending on its origin: the shorter, and more energy-efficient pathway where zooplankton directly graze on phytoplankton, and the longer, more inefficient bacteria–bacterivorous pathway with additional intermediate trophic levels, due to the small size of bacteria (Berglund et al. 2007; Faithfull et al. 2012). To estimate food chain length we used the ratio of PP to total BP (PP:BP ratio), with a higher PP:BP indicating more efficient and shorter energy transfer pathways via PP (Jansson et al. 2000; Sommer and Sommer 2006). Higher DOC concentrations are associated with higher BP relative to PP, wherefore an increase in DOC promotes longer food chains (Tranvik 1988; Kritzberg et al. 2006; Berglund et al. 2007). Higher N availability in N limited boreal lakes on the other hand should increase the relative contribution of PP to PEM, resulting in increased food quantity, and a shorter and more energy efficient pathway to crustacean zooplankton via PP.

Food web efficiency (FWE = zooplankton production per total pelagic energy mobilized) denotes how efficiently energy is transferred from basal trophic levels (phytoplankton and bacteria) to zooplankton (Berglund et al. 2007). FWE depends both on the length of the food chain (the PP:BP ratio) and on how efficiently energy is transferred between each trophic link. The latter can be affected by organism stoichiometry, i.e. changes in phytoplankton N:P, carbon (C):P ratios, which are determined by light and nutrient availability (Sterner et al. 1998; Elser et al. 2009a). Enhanced N availability in N-limited lakes should, therefore, stimulate growth (C production) and promote phytoplankton with high C:P and N:P ratios, potentially resulting in poor quality food for crustacean consumers, especially those with high P demands (Sterner et al. 1998; Elser et al. 2009a). Reduced phytoplankton food quality may counteract the positive effects of increased N availability on food quantity (PEM) and increased PP:BP for zooplankton. Even more difficult to predict are how responses of energy transfer efficiencies to increased N availability will differ between lakes with different DOC concentrations (Hessen 2013; Solomon et al. 2015).

The aim of this study was to assess the effects of enhanced inorganic N availability on total pelagic energy mobilization (PEM, i.e. food quantity), food chain length (i.e. the PP:BP ratio), and food quality (i.e. seston N:P and C:P stoichiometry), and the consequences for zooplankton growth and FWE in unproductive N-limited boreal lakes across a DOC gradient. For this reason we conducted whole lake inorganic N enrichment experiments in three lake pairs (one control, one N enriched) with varying DOC levels (low, medium, high). In each experimental lake, we performed in situ mesocosm experiments (in triplicates) in late summer in 2011 (before) and 2013 (second year of fertilization) to assess zooplankton production in fish-free environments before and after enrichment. We hypothesize that N fertilization will

1. Increase food quantity (PEM) by reducing N limitation of phytoplankton, promote shorter food chains (increase PP:BP) by increasing PP, and decrease phytoplankton food quality by increasing seston C:P, N:P ratios in all fertilized lakes. The size of these effects will decrease with increasing DOC concentration due to increasing light limitation for primary producers.
2. Zooplankton growth and FWE will increase in response to N fertilization due to higher PP and the promotion of shorter food chains (increased PP:BP). Thus, we expect that increased food availability and shorter food chains resulting from N fertilization will counteract any negative effects on zooplankton caused by reduced phytoplankton food quality (i.e. enhanced seston C:P and N:P ratios).
3. Last, we predict that DOC will reduce the response of zooplankton growth and FWE to N fertilization due to light limitation of primary producers.

## Materials and methods

### Experimental setup

Six lakes of similar size and depth (Table 1), with small littoral zones, were chosen as experimental lakes in northern boreal Sweden (64.12–64.25°N, 18.76–18.80°E). The catchment areas consisted of coniferous forests and open *Sphagnum* sp. dominated mires. The lakes are typically ice covered from early November to early May. Thermal stratification develops from mid to late May until mid to late September. Atmospheric N deposition is low (wet dissolved inorganic N deposition <200 kg km^−2^ year^−1^) (Bergström et al. 2008), and except for forestry, anthropogenic influences on the lakes are minimal.

**Table 1:** Physical and chemical characteristics of the epilimnion in the experimental lakes (control lakes; N-lakes) in the reference year (2011) during the investigated timeframe (June–August)

Parameters	Control lakes			N-lakes		
	Nästjärn (Low DOC)	Mångstenstjärn (Med. DOC)	Övre Björntjärn (High DOC)	Fisklösan (Low DOC)	Lapptjärn (Med.DOC)	Nedre Björntjärn (High DOC)
Catchment area (ha)	3.4	14.1	284.0	8.9	16.8	324.9
Surface area (ha)	1.0	1.8	5.0	1.7	2.0	3.2
Mean depth (m)	4.2	5.3	4	2.1	2.5	6
Max depth (m)	10.4	9.7	8	7.8	6.5	9.7
Epilimnion depth (m)	1.3	1.0	1.1	1.9	1.1	1.0
% Epilimnion	59	51	65	86	75	51
DOC_epi_ (mg L^−1^ ± SD)	6.9 ± 0.2	10.1 ± 0.2	21.0 ± 6.2	6.9 ± 0.4	11.4 ± 0.5	18.2 ± 3.9
TP (µg L^−1^ ± SD)	9.8 ± 2.6	12.0 ± 1.9	18.5 ± 2.8	8.6 ± 1.4	11.0 ± 3.0	17.9 ± 4.3
TN (µgL^−1^ ± SD)	240 ± 63	324 ± 60	476 ± 63	229 ± 38	333 ± 41	439 ± 59
DIN (µg L^−1^ ± SD)	7.7 ± 4.3	10.2 ± 4.5	17.8 ± 6.2	4.3 ± 2.2	9.6 ± 4.6	17.4 ± 5.8
DIN load natural (g m^−2^ year^−1^)	0.03	0.15	1.02	0.05	0.07	1.40
DIN load artificial (g m^−2^ year^−1^)	0.0	0.0	0.0	1.0	1.1	1.8
Temperature_epi_ (°C ± SD)	18.0 ± 3.3	17.5 ± 2.8	16.5 ± 2.6	17.3 ± 3.4	17.8 ± 3.1	16.6 ± 2.5
Light (*I*_m_ ± SD)	0.17 ± 0.03	0.09 ± 0.01	0.06 ± 0.01	0.37 ± 0.05	0.17 ± 0.02	0.04 ± 0.00
Light (*k*_d_ ± SD)	1.3 ± 0.2	2.1 ± 0.4	4.2 ± 0.9	1.1 ± 0.2	2.2 ± 0.4	4.1 ± 0.5

Mean values (*n* = 8) are presented followed by standard deviations (SD). % Epilimnion shows the percentage contribution of the epilimnion to the whole lake volume. *I*_m_ is the mean irradiance for the mixed water layer, whereas *k*_d_ is the vertical attenuation coefficient for PAR (in m^−1^)

*DOC* dissolved organic carbon, *TP* total phosphorus, *TN* total nitrogen, *DIN* dissolved inorganic nitrogen, *Med.DOC* medium DOC concentration

Lakes were selected along a gradient of DOC concentration with one lake pair at each DOC level (Low DOC ~7 mg L^−1^, Medium DOC ~11 mg L^−1^, High DOC ~20 mg L^−1^, Table 1), which represents the typical variety of oligotrophic lakes in the boreal landscape (Downing et al. 2006; Sobek et al. 2007). Lakes covered a range in water retention times (WRT) with longer WRT in low DOC lakes (740–810 days) and shorter WRT in high DOC lakes (40–50 days). For each DOC level, one lake served as a control lake and the other lake was fertilized with N. The study reference year was 2011 (Before; all lakes), and 2012 and 2013 were the impact years (After, with N fertilization in 2012 and 2013). Fish communities were similar within each lake pair; the low DOC lakes were fishless, the medium DOC lakes had stunted perch populations, and the high DOC lakes had normally size distributed perch populations. Here, we assess results from mesocosm experiments and lake monitoring (explained in detail below), where we compare data from 01-Aug to 25-Aug in 2011 (*n* = 3; reference year), with data from 22-Jul to 05-Aug in 2013 (*n* = 3; impact year; second year of treatment), to explicitly assess how inorganic N enrichment affects zooplankton growth in the experimental lakes.

Nitrogen in the form of dissolved potassium nitrate (14 M N as KNO_3_) in 2012 and concentrated nitric acid (14 M N as HNO_3_) in 2013 were evenly distributed across the surface of the N fertilized lakes. Different sources of N were used in 2012 and 2013 due to practical reasons, as HNO_3_ turned out to be easier to dilute in lake water than KNO_3_. Nitrate (NO_3_^−^) was used, as NO_3_^−^ leakage from the catchment is the most typical N compound entering boreal lakes originating from atmospheric N deposition and forest clear cutting (Moldan et al. 2006; Kreutzweiser et al. 2008). As leaching events typically follow high catchment runoff during winter and spring (Bergström et al. 2008), seasonal variation in external inorganic N loading was mimicked by fertilizing the whole water column once during ice cover in 2012 (late March) and 2013 (early April). For the rest of the growing season, nitrate was added from the onset of stratification in late May/early June until late August. Nitrate was added to increase the dissolved inorganic nitrogen (DIN) in the whole lake (during ice off) or in the epilimnion by 100 µg N L^−1^, to mimic inorganic N inputs for lakes in southwest Sweden with high N deposition (Bergström et al. 2008). The amount of fertilizer added to each N-lake was calculated depending on the lake volume, stratification depth and the theoretical water residence time in order to increase DIN loads in all lakes to the same extent. Thus, during stratification, fertilization occurred every second week in all lakes in 2012. In 2013, N fertilization was performed every second week in the low and medium DOC N-lakes, whereas in the high DOC N-lake, fertilization occurred every week due to a shorter water residence time. In total, we added an amount of 1–1.8 g N m^−2^ year^−1^ (Table 1) which equals the atmospheric DIN loads in southwestern Sweden from 2011–2014, being 3–4 times as high as in our study area (southwestern Sweden: 0.8–1.7 g N m^−2^ year^−1^; our study area: 0.3–0.4 g N m^−2^ year^−1^) (SMHI 2016).

### Mesocosm experiment

In order to measure zooplankton production independent of fish predation, we constructed mesh cubic mesocosms (1 m^3^; 104 µm nylon mesh net) which allowed a natural flow of water in and out of the mesocosms while keeping fish and invertebrate predators (mainly *Chaoborus* larvae) out. Additionally, zooplankton biomass and community composition were assessed from the actual lakes (i.e. outside mesocosms).

Mesocosms were deployed at the surface in each lake, at water depths of approx. 2 m. Floats were attached to the top, and the mesocosms were weighted and anchored at the corners to keep them upright and in location. One top side corner was secured with Velcro^©^ binding so they could be opened for sampling. In 2011 (reference year), three mesocosms per lake were suspended in all six lakes on the 28-Jul and 29-Jul (i.e. in total 18 mesocosms). Crustacean zooplankton collected from the deepest point of the lake on 01-Aug-2011 (day 0) were added to each mesocosm at ambient lake densities after removing zooplankton predators. On the same day (day 0) a subsample for zooplankton initial biomass and community composition was taken from the deepest point of the lake using the same 100 µm mesh net used for inoculation of zooplankton in the mesocosms. Zooplankton samples were first washed in 90% ethanol and then preserved with 70% ethanol. On 11-Aug (day 10) and 25-Aug (day 24) in 2011, zooplankton production was estimated from samples taken by hauling a 100-µm net from the bottom of each mesocosm to the top (details on production assessment see below). On the final experimental day (day 24) an additional sample of lake zooplankton biomass and community composition was taken from the deepest point of the lake. All zooplankton samples were preserved in ethanol as described above and kept dark at 6 °C until further analysis. In 2013 (impact year 2), triplicate mesocosms were deployed on 22-Jul in the same manner as 2011 in all six experimental lakes. Crustacean zooplankton were added to each mesocosm on 22-Jul (day 0). In 2013, subsequent zooplankton samplings occurred on 30-Jul (day 8) and 05-Aug (day 14). Crustacean zooplankton taxa were identified, counted and measured using inverted microscopy (100× magnification), and the image analysis system Image Pro Plus 6.2. High magnification was used since the presence of egg-bearing females and the number of eggs per female for all taxa was assessed from the mesocosm samples in order to estimate zooplankton production (see below). The length of all individuals was measured and length–weight regressions (Bottrell et al. 1976) and a conversion factor of 0.48 C dry weight^−1^ (Andersen and Hessen 1991) were used to calculate zooplankton carbon biomass.

### Sampling

Regular sampling of physical, chemical, and biological parameters was conducted every second week during the growing season at the deepest point in each lake. Here, we use data from three sampling occasions that were performed at the same time as the mesocosm experiments were conducted, i.e. in 2011 (1-Aug, 11-Aug, 25-Aug) and 2013 (22-Jul, 29-Jul, 05-Aug). Temperature (Temp) and photosynthetically active radiation (PAR) profiles were measured in the lakes using handheld probes (Temp, O_2_: YSI ProODO; PAR: LI-193 Spherical Quantum Sensor/LI-COR Biosciences). The light extinction coefficient (*k*_d_) was calculated from PAR profiles following the procedure described in (Wetzel 2001). Further, values for daily LUX insolation were measured using light loggers (HOBO UA-002-64, 10 min logging interval) on the open shore of each lake. Composite samples for chemical and biological parameters were taken from the mid epilimnion using a Ruttner sampler. Subsamples were taken from the composite samples for analyses of water chemistry, seston stoichiometry (C:P, N:P) and total bacterial production (BP).

### Chemical analysis

Water samples were analyzed for DOC, dissolved inorganic carbon (DIC), ammonium (NH_4_^+^), nitrite + nitrate (NO_2_^−^ + NO_3_^−^), total nitrogen (TN) and total phosphorus (TP). Dissolved inorganic N (DIN) was estimated as: NH_4_^+^ + NO_2_^−^ + NO_3_^−^. For DOC determination, samples were filtered through pre combusted (450 °C, 5 h) Whatman GF/F filters, acidified (1.2 M HCl) and kept in the dark at 6 °C until analysis using a HACH-IL 550 TOC-TN analyzer (Hach-Lange GmbH Düsseldorf, Germany). For DIC analyses, 4 mL water was injected into gas-tight glass vials (22 mL; PerkinElmer Inc., US) containing 50 µL 1.2 M HCl and N_2_. CO_2_ concentrations in the vial headspace were analyzed using a gas chromatographer (Clarus 500, Perkin Elmer Inc., US) equipped with a flame ionization detector. For N and P, samples were kept frozen until analysis which was performed following descriptions elsewhere (Bergström et al. 2013). For DIN analysis (i.e. NO_2_^−^ + NO_3_^−^, and NH_4_^+^), samples were filtered through 0.45 µm cellulose acetate filters. TN was analyzed using a HACH-IL 550 TOC-TN analyzer (Hach-Lange GmbH Düsseldorf, Germany) and TP using a JASCO V-560 spectrophotometer (Easton, Maryland, USA) after applying the molybdenum blue method following Bergström et al. (2013). DIN:TP and TN:TP ratios are presented as molar ratios. All chemical analyses were performed at the Department of Ecology and Environmental Science (EMG), Umeå University. Edible seston (<50 µm) stoichiometry was determined by filtering known volumes of pre filtered (50 µm mesh) epilimnion water onto GF/F filters for analysis of particulate C and N (pre combusted filters at 550 °C, 4 h) and P (acid washed filters, 1.2 M HCl). Particulate organic C and N were measured using a Costech ECS 4010 elemental analyzer (Costech International S. P. A.) at the Limnology Department, Uppsala University, Sweden (Bergström et al. 2015). Filters for particulate P were analyzed as for TP (cf. above) at Umeå University (EMG).

### Bacteria and phytoplankton production

BP in the epilimnion was measured using the [^3^H]-leucine incorporation method (Smith and Azam 1992), following the protocol in Karlsson et al. (2002). Triplicate 1.2 mL aliquots of sample and one trichloracedic acid (TCA) killed control were incubated with 8 µL leucine isotope (specific activity 3.9 TBq mmol; PerkinElmer, Boston) for 60 min in darkness at in situ temperatures. The incubation was ended by adding 65 µL 100% TCA, and the ^3^H activity was measured with a scintillation counter (Beckman LS 6500).

Volumetric lake PP was measured using the ^14^C incorporation method (Schindler et al. 1972). Water samples were taken with a Ruttner sampler from 5 to 7 steps (the uppermost samples at depths of: 0; 0.2; 0.5; 1; 1.5 m) in a depth profile from the surface down to the depth corresponding to 1% of surface irradiance based on in situ light measurements. Duplicate samples were measured down to 1 m. Dark control bottles were added at the surface, at 1 m, and at the lowest light level. Samples were incubated at sampling depth in 125 mL borosilicate glass bottles after adding 3 µL of ^14^CHNaO_3_ (37 MBq mL^−1^) (PerkinElmer, Boston). All bottles were incubated for 4 h over midday. Bottles were kept dark during transport to the laboratory. Aliquots of 5 mL per bottle were placed in scintillation vials and acidified with 50 µL 1.2 M HCl to end the reaction. Samples were then shaken and aerated for 24 h to remove residual inorganic ^14^C and carbonates. A scintillation cocktail (10 mL Optiphase ‘Hisafe’ 3 multipurpose) was added to each sample, and ^14^C activity measured using a Beckman-coulter LS6500 scintillation counter. Measured PP was converted to daily rates using the ratio of photosynthetically active irradiance during the incubation period to whole day irradiance (Karlsson et al. 2002). The PP:BP ratio (volumetric) was calculated for each sampling occasion using volumetric PP and BP and is used as an indicator of food chain length (Jansson et al. 2000; Berglund et al. 2007).

### Pelagic energy mobilization and zooplankton production

Total pelagic energy mobilization (PEM) in the epilimnion for the experimental periods (*n* = 3) in 2011 (01-Aug to 25-Aug; reference year) and 2013 (22-Jul to 05-Aug; impact year 2) was calculated as

$${\text{PEM}} = {\text{BP}} + {\text{PP}}.$$

Volumetric zooplankton production for cladocerans was calculated according to Bottrell et al. (1976), Mason and Abdulhussein (1991), and Dickman et al. (2008) using changes in cladoceran biomass and egg counts per female over time, and egg development times were estimated according to temperature. The copepod production (calanoids and cyclopoids counted separately) was calculated as the sum of production for eggs, nauplii, and copepodites:

$${\text{Copepod production}} = \frac{{N_{\text{e}} \times W_{\text{e}} }}{{T_{\text{e}} }} + \frac{{N_{\text{n}} \times W_{\text{n}} }}{{T_{\text{n}} }} + \frac{{N_{\text{c}} \times W_{\text{c}} }}{{T_{\text{c}} }},$$

where *N*_e_ is the density of eggs (eggs; L^−1^), *N*_n_ is the density of nauplii (nauplii; L^−1^) and *N*_c_ is the density of copepodites (copepodites; L^−1^). *W* is the weight increase at each stage (g) and *T* is the duration of each stage (days). The remainder of the method followed the protocol of Dickman et al. (2008). Total zooplankton production was calculated as the sum of production of each major zooplankton taxon, i.e. *Bosmina* spp. *Holopedium gibberum*, *Ceriodaphnia* spp., *Polyphemus* spp., *Diaphanosoma* spp., *Daphnia* sp., *Cyclopoida* sp. and *Calanoida* sp.

Food web efficiency (FWE, dimensionless) was calculated as follows:

$${\text{FWE}} = \frac{\text{Total zooplankton production}}{\text{Pelagic energy mobilization}}.$$

### Statistics

To test the effects of ‘DOC concentration’ and ‘year’ on response variables in non-manipulated lakes, data from all lakes in 2011 plus the control lakes in 2013 were used during the time frame of the mesocosm experiment (July–August). Data were analyzed using repeated measures two-way ANOVA with ‘DOC concentration’ and ‘year’ as explanatory variables and ‘date’ nested in ‘lake’ as a random effect to correct for pseudoreplication over time and individual lake effects. To analyze N effects we calculated the net change (Δ) of each variable as 2011–2013 for each lake and variable separately. The change (i.e. ΔPP; ΔBP; ΔPEM; Δ  %PEM_PP_; ΔSeston N:P; ΔSeston C:P) in each variable was then analyzed using mixed effect model ANOVAs with ‘N enrichment’ as an explanatory variable and ‘date’ as a random effect to correct for pseudoreplication over time. Standardized effect sizes were calculated using Cohens *d*, where |*d*| < 0.2 is considered a weak effect and |*d*| > 0.8 is considered a strong effect. To explore whether DOC, year, N fertilization, or their interaction affected the zooplankton community composition, we applied permutational multivariate analysis of variance (PERMANOVA) analysis on zooplankton community composition data on species data [log(*x* + 1)-transformed]. Distances among the samples were computed as Bray–Curtis dissimilarities. We evaluated how seston C:P and N:P ratio differed with DOC concentration in non-manipulated and fertilized lakes using linear regression analysis of yearly means (non-manipulated lakes: *N* = 12, fertilized lakes: *N* = 6) over the growing season (June–September). Further, we used linear regression to determine effects of seston stoichiometry on TZP and FWE using data from all lakes over the duration of the mesocosm experiment in both 2011 and 2013 (*n* = 24). Prior to analysis all data were tested for normality of distributions and variances and data were transformed as appropriate if necessary. All statistical analyses were performed in the statistical program R (R Development Core Team; version 3.1.2), using the package “nlme” for mixed model analysis (Pinheiro et al. 2009) and “vegan” for the analysis of zooplankton community composition (Oksanen et al. 2016).

## Results

### Background conditions

In non-manipulated lakes (control lakes all years, and N lakes in 2011) light extinction, TN and TP concentrations were higher in lakes with high DOC (Tables 1, 2). In 2011 across all lakes, average growing season epilimnion light availability, precipitation, and flushing rates were twice as high as in 2013. N fertilization caused a sixfold increase in DIN concentration and an eightfold increase in DIN:TP (Table 3). TN, TN:TP and TP parameters did not show any significant responses to fertilization during the whole lake experiment (Table 3). During the mesocosm experiments (late July to late August) epilimnion temperatures were similar in 2011 and 2013 (seasonal mean 17 °C). Light conditions during the mesocosm experiment reflect the same differences as over the whole growing season in 2011 and 2013 (June–September). All data presented below were collected during mesocosm experiments (i.e. late July to late August, 2011 and 2013), if not stated otherwise.

**Table 2:** Linear regression of response variables (Response) and explanatory variables (Expl.) for yearly means of unfertilized lakes in 2011 and 2013. (No fert., *n* = 12), fertilized lakes (fert., *n* = 6), and all lakes (all, *n* = 24)

Treatment	Expl.	Response	Formula	*R* ^2^	*p*
No fert.	DOC	*k*_d_	−0.11 + 0.20 (DOC)	0.72	**<0.001**
		TN	206 + 13 (DOC)	0.55	**<0.001**
		TP	4.0 + 0.7 (DOC)	0.63	**<0.001**
		Seston N:P	36.9−0.3 (DOC)	0.21	0.130
		Seston C:P	327−4 (DOC)	0.32	0.056
		Seston C	513−9 (DOC)	0.39	**0.039**
Fert.		Seston N:P	83.4−2.7 (DOC)	0.66	**0.049**
		Seston C:P	821−32 (DOC)	0.86	**0.008**
		Seston C	1451−60 (DOC)	0.93	**0.002**
All	Seston N:P	_log_TZP	3.1−1.6 (_log_N:P)	0.19	**0.033**
		_log_FWE	2.1−1.8 (_log_N:P	0.23	**0.019**
	Seston C:P	_log_TZP	4.3−1.4 (_log_C:P)	0.19	**0.035**
		FWE	3.3−1.6 (_log_C:P)	0.21	**0.026**

Significant *p* values are shown in bold (*p* < 0.05)

**Table 3:** Chemical parameters in the epilimnion for the experimental lakes (means of control and N-lakes) during the investigated time frame (June–August, *n* = 7) before (2011) and after N fertilization (2012; 2013, pooled)

Parameters	Control lakes		N-lakes		*p*	*F*_*df* = 1,4_
	Before (mean ± SE)	After (mean ± SE)	Before (mean ± SE)	After (mean ± SE)		
TN (µg L^−1^)	356 ± 25	391 ± 13	349 ± 21	499 ± 32	0.194	2.43
TP (µg L^−1^)	13.8 ± 1.0	13.8 ± 0.8	13.1 ± 1.1	13.1 ± 0.9	0.432	0.76
DIN (µg L^−1^)	12.3 ± 1.4	11.5 ± 1.3	11.3 ± 1.5	69.6 ± 8.2	0.009*	22.43
TN:TP (molar)	59 ± 4	74 ± 6	62 ± 3	106 ± 12	0.443	0.73
DIN:TP (molar)	1.9 ± 0.2	2.0 ± 0.3	1.8 ± 0.1	14.3 ± 1.7	0.027*	11.60

Mean values are presented followed by standard errors (± SE)

*TN* total nitrogen, *TP* total phosphorus, *DIN* dissolved inorganic nitrogen

Asterisk indicates significant fertilization effects (*p* < 0.05) for ‘ΔAfter’ (difference between respective value before and after enrichment compared to the difference in the control lakes)

### Basal production

During the mesocosm experiments, PP in non-manipulated lakes (i.e. control lakes: all years; N-lakes: 2011) did not differ across the DOC gradient. In the fertilized lakes, N fertilization doubled PP in 2013 (Table 4). Further, the N enrichment effects differed with DOC concentration and were strongest in low and medium DOC lakes (Table 4).

**Table 4:** Effects of explanatory variables on biotic response variables

Parameters	DOC		Year		Year:DOC		N effect		N:DOC				
	*F*_*df* = 2,3_	*p*	*F*_1,9_	*p*	*F*_2,9_	*p*	*F*_1,5_	*p*	*F*_2,5_	*p*	ES (Cohens *d*)		
											Low	Med	High
PP	2.16	0.263	**8.65**	0.017*	**6.49**	0.018*	**63.70**	<0.001*	**10.86**	0.015*	**6.2**	**5.1**	**1.6**
BP	2.74	0.211	0.00	0.963	**6.05**	0.022*	2.28	0.191	**8.41**	0.025*	−**7.6**	**1.8**	**2.9**
PEM	0.46	0.670	4.42	0.065	0.30	0.748	**165.80**	<0.001*	**7.75**	0.029*	**4.4**	**16.0**	**20.7**
PP:BP	5.40	0.101	**5.88**	0.038*	**11.47**	0.003*	**22.07**	0.005*	**15.62**	0.007*	**6.4**	**0.8**	−**0.2**
Seston N:P	0.06	0.946	1.20	0.302	1.50	0.274	**11.51**	0.019*	0.14	0.874	1.2	1.7	13.0
Seston C:P	0.03	0.971	0.03	0.860	3.24	0.087	1.13	0.336	0.52	0.624	0.8	1.4	−0.2
TZP	2.61	0.221	**44.63**	<0.0001*	0.23	0.797	1.25	0.274	**4.17**	0.027*	−**2.8**	−**0.4**	**0.6**
Calanoid prod.	5.80	0.093	**80.52**	<0.0001*	1.08	0.353	0.05	0.830	**4.20**	0.027*	−**2.2**	**0.1**	**0.7**
Cyclopoid prod.	5.92	0.091	2.51	0.122	0.73	0.492	**16.53**	<0.001*	**4.20**	0.027*	−**1.6**	−**1.8**	**0.1**
*Bosmina* prod.	0.94	0.481	**7.75**	0.009*	**8.23**	0.001*	0.01	0.912	2.23	0.128	−0.7	0.7	0.5
*Ceriod.* prod.	**41.18**	0.007*	0.05	0.832	1.25	0.301	0.65	0.428	0.38	0.685	0.3	−0.3	−0.1
FWE	1.73	0.316	**38.13**	<0.001*	0.17	0.847	**5.63**	0.026*	**7.08**	0.004*	−**2.3**	−**1.1**	**0.6**

Explanatory variables were DOC level (DOC), year, the interaction of year and DOC (Year:DOC), N fertilization (N effect), and the interaction of DOC with N fertilization (N:DOC). Given are *F*_*df* = degrees of freedom_, *p* values (*p* < 0.05 marked with asterisk and in bold), and effect sizes for the interaction N:DOC (ES, Cohens *d*) for each DOC level (low, medium, high)

Note that N effects which increase the respective response variable are indicated by positive ES values and N effects that decrease the respective response variable by negative ES values

**Fig. 2:**
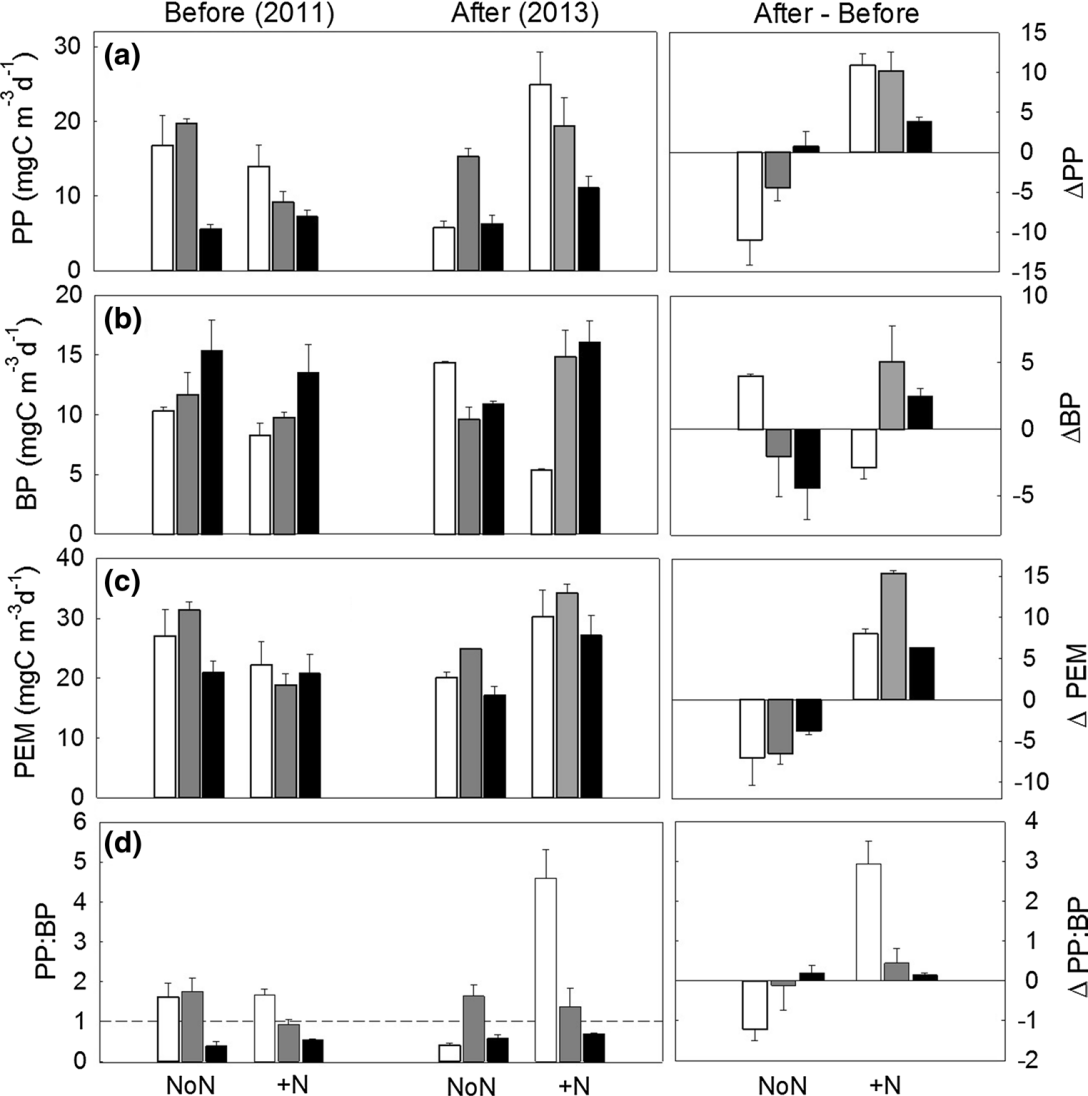
Volumetric estimates (± standard errors) of **a** primary production (PP), **b** total bacterial production (BP), **c** total pelagic energy mobilization (PEM = PP + BP), and **d** food chain length shown as PP:BP during the mesocosm experiment (July–August, *n* = 3) in the control lakes (NoN) and N lakes (+N) before (‘Before’, 2011), and after N fertilization (‘After’, 2013) across the DOC gradient (*white* low DOC, *gray* medium DOC, *black* high DOC). Fertilization effects are presented as ‘Δ After’, illustrating the difference between respective values before and after N fertilization

The BP in non-manipulated lakes did not differ between lakes with different DOC concentrations (Fig. 2b; Table 4). N fertilization effects differed with DOC concentrations: N fertilization had a strong negative effect on BP in the low DOC lake, whereas it had strong positive effects on BP in medium and high DOC lakes (Table 4).

The PEM was stable across the DOC gradient (Fig. 2c). N fertilization caused a 50% increase in PEM in N-lakes in 2013, whereas PEM in control lakes decreased by 20% in 2013 (Table 4).

The PP:BP in non-manipulated lakes did not differ across the DOC gradient. N fertilization caused a threefold increase in the PP:BP ratio (Fig. 2e; Table 4). N effects on PP:BP were strongest in the low DOC lake and decreased with increasing DOC concentrations (Table 4).

### Seston stoichiometry

**Fig. 3:**
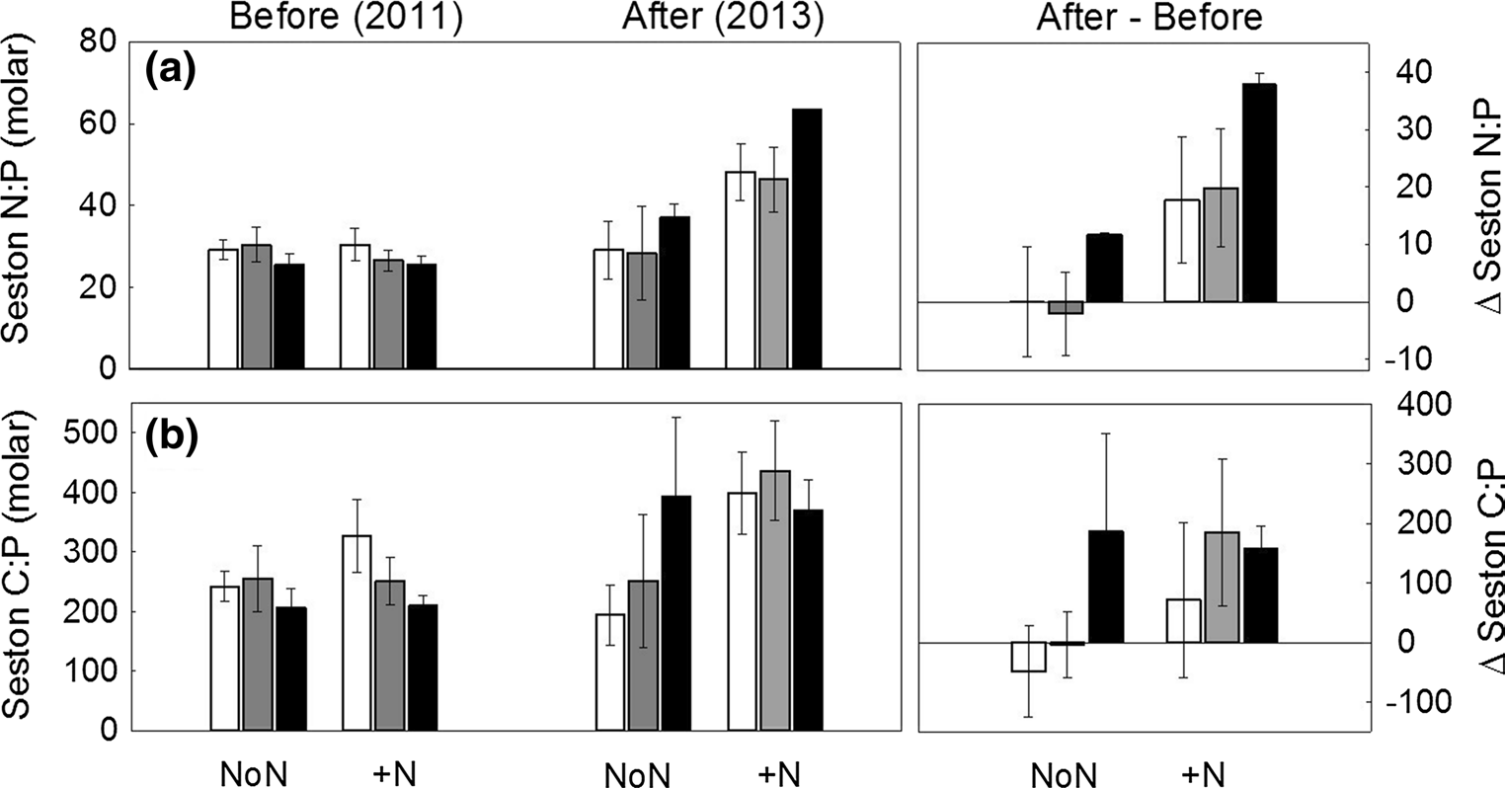
Seston stoichiometry (± standard errors) of **a** N:P, and **b** C:P molar ratios during the mesocosm experiment (July–August, *n* = 3) in control lakes (NoN) and N lakes (+N) before (‘Before’, 2011), and after N fertilization (‘After’, 2013) across the DOC gradient (*white* low DOC, *gray* medium DOC, *black* high DOC). Fertilization effects are presented as ‘Δ After’, illustrating the difference between respective values before and after N fertilization

**Fig. 4:**
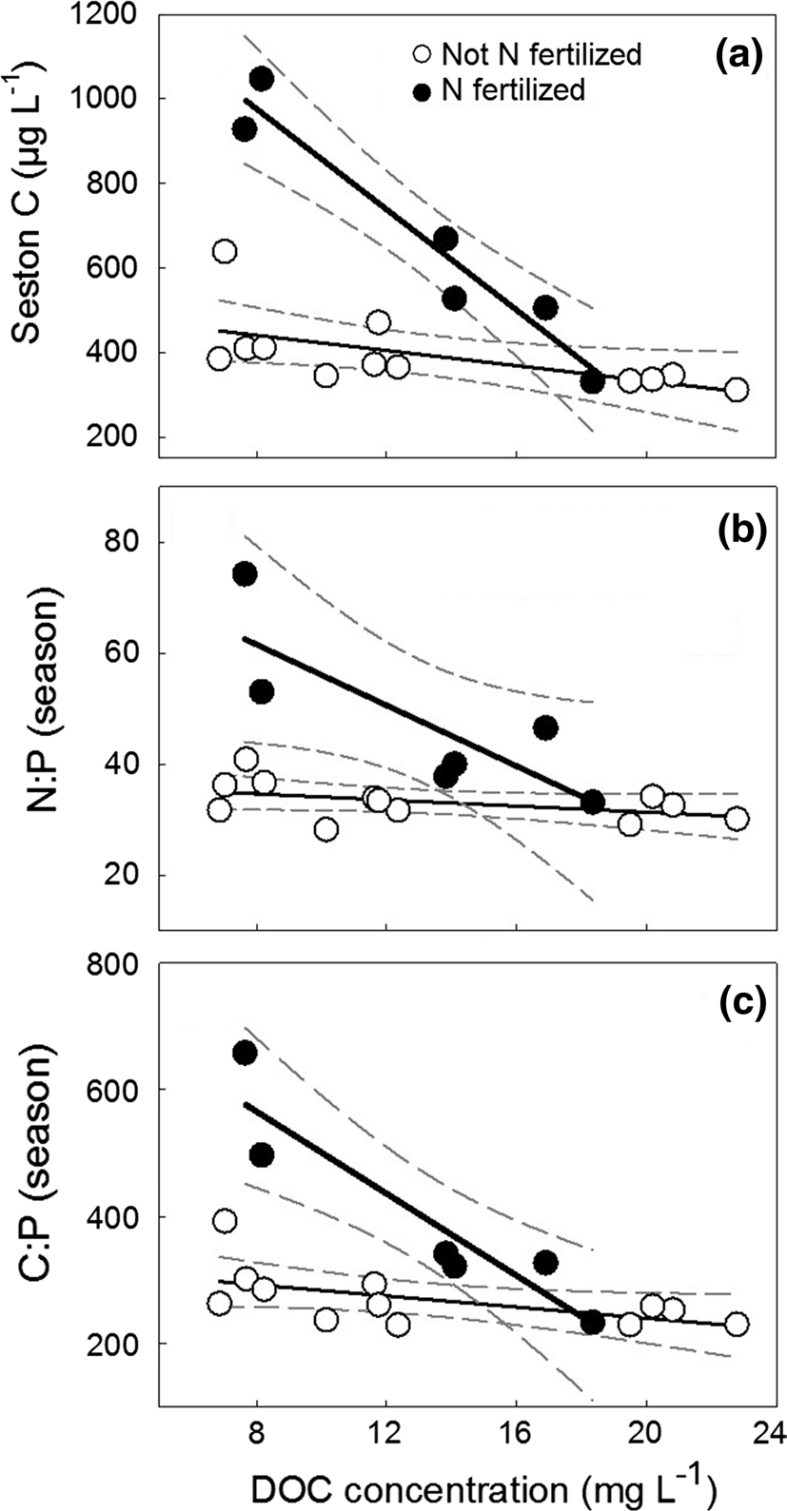
Changes in **a** seston carbon (µg L^−1^), **b** seston N:P and **c** C:P ratios during the whole growing season in response to N fertilization in lakes across the DOC gradient (*black* fertilized lakes, *n* = 6, i.e. N lakes in 2012, 2013; *white* non-manipulated lakes, *n* = 12, i.e. control lakes in all years, N lakes in 2011) with regression lines (*thick line* fertilized lakes, *thin line* non-manipulated lakes) and 95% confidence intervals (*dashed*)

In non-manipulated lakes, the seston N:P and C:P ratios were stable between years and across the DOC gradient (Fig. 3, Tables 2, 4). N fertilization resulted in a significant increase in seston N:P ratios across the DOC gradient with average N:P increasing from 28 to 50. The seston C:P ratios did not show a significant increase with N addition, although average C:P increased from 247 to 403 and C:P showed an increasing trend in low and medium DOC lakes after N fertilization (Table 4). Further, the threshold elemental C:P ratio of 300, which can indicate P limitation in *Daphnia* (Sterner and Elser 2002), was exceeded in all N-lakes during the mesocosm experiment, and only twice in the control lakes (once in low, and once in the medium DOC lake). Last, data over the whole growing season (yearly means June–September) showed that the N effect on seston N:P, C:P and seston C decreased with increasing DOC (Fig. 4; Table 2).

### Crustacean zooplankton community composition

**Fig. 5:**
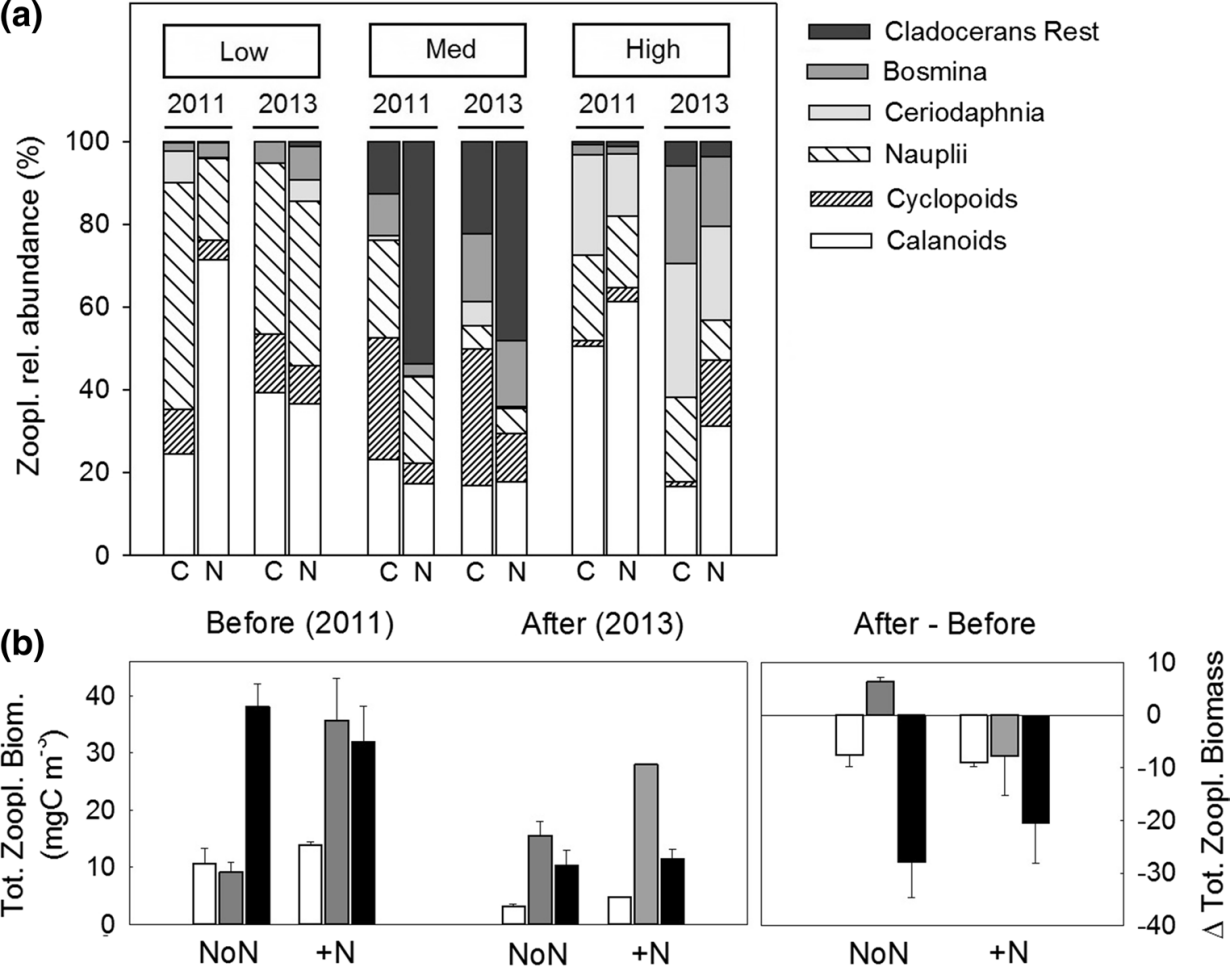
**a** Community composition of zooplankton, and **b** total zooplankton biomass (± standard errors) during the mesocosm experiment (July–August, *n* = 3) in control lakes (NoN), N lakes (+N) before (‘Before’, 2011), and after N fertilization (‘After’, 2013) across the DOC gradient. Fertilization effects are presented as ‘Δ After’, illustrating the difference between respective values before and after N fertilization

Lake zooplankton communities (i.e. sampled outside the mesocosms) differed with DOC and between years (PERMANOVA, DOC: *p* = 0.001, *F*_2_ = 13.12, *R* ^2^ = 0.45; year: *p* = 0.001, *F*_1_ = 9.02, *R* ^2^ = 0.16) (Fig. 5a). Specifically, DOC was important for cyclopoids and the cladoceran *Ceriodaphnia,* which showed highest biomasses at medium and high DOC, respectively (Table 4). The year effect was important for both copepod groups. In control lakes, calanoid biomass was fivefold lower in 2013 than in 2011 (Table 4) and cyclopoid biomass was 44 and 73% lower in low and high DOC lakes, respectively. However, cyclopoid biomass in the medium DOC lakes doubled in 2013 (Table 4). Additional variation in community composition can be explained by lake-specific characteristics (i.e. Lake ID; *R* ^2^ = 0.05, *F*_1_ = 1.54, *p* = 0.001). The N fertilization did not affect zooplankton community composition. Total zooplankton biomass was stable between years and across the DOC gradient (Fig. 5b), and N fertilization did not affect total zooplankton biomass or biomass of any zooplankton taxa specifically.

### Zooplankton production

**Fig. 6:**
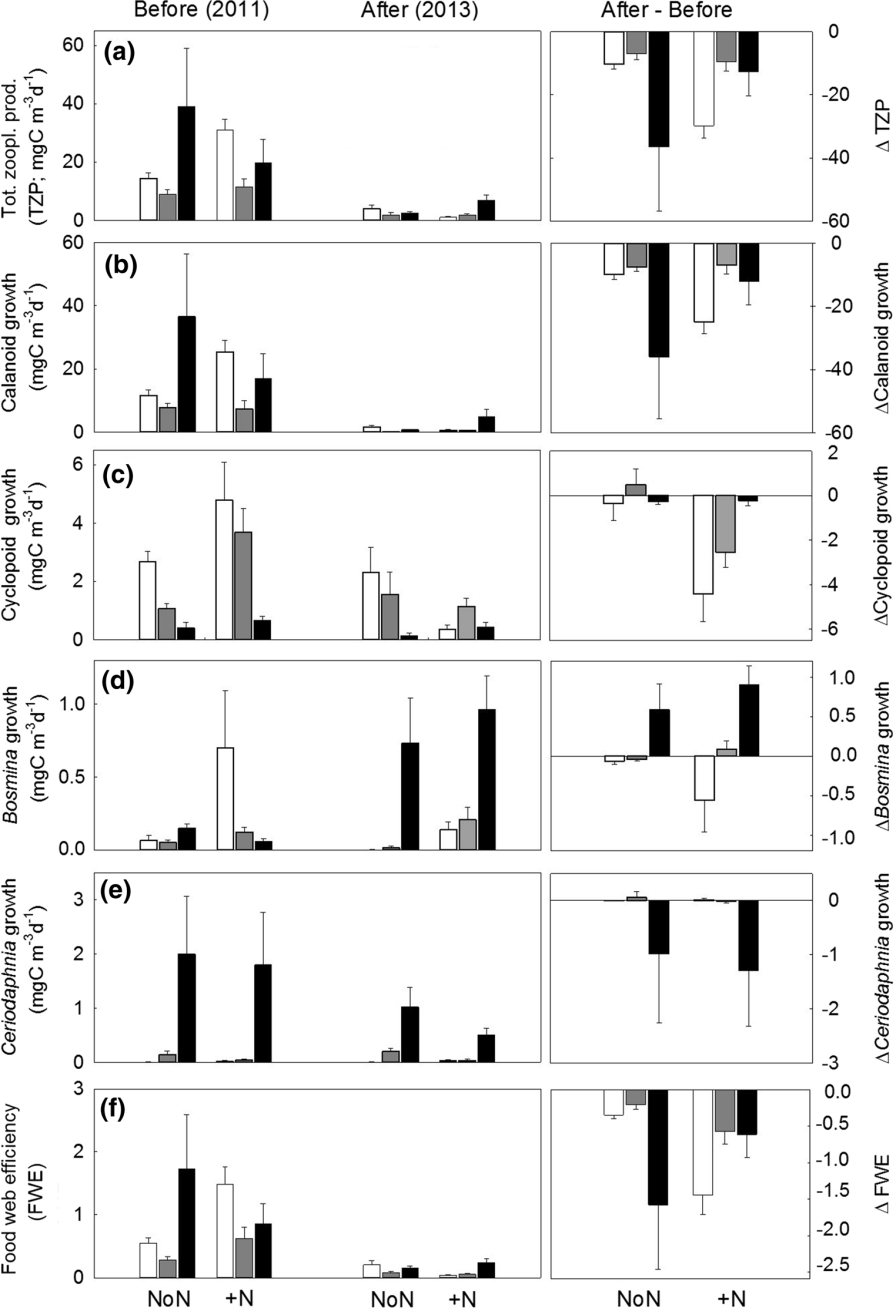
Volumetric estimates (± standard errors) of **a** total zooplankton production (TZP), growth rates of **b** *Calanoida*, **c** *Cyclopoida,* **d** *Bosmina*, and **e** *Ceriodaphnia*, and **f** food web efficiency (FWE) during the mesocosm experiment (July–August, *n* = 2) in control lakes (NoN) and N lakes (+N) before (‘Before’, 2011), and after N fertilization (‘After’, 2013) across the DOC gradient (*white* low DOC, *gray* medium DOC, *black* high DOC). Fertilization effects are presented as ‘Δ After’, illustrating the difference between respective values before and after N fertilization

In the control lakes, total zooplankton production (TZP) in the mesocosms was sevenfold lower in 2013 than in 2011 (Fig. 6a; Table 4). TZP did not differ between control lakes with different DOC concentrations. However, N fertilization had a strong negative effect on TZP in the low DOC lake, a weak negative effect in the medium DOC lake, and N fertilization increased TZP in the high DOC lake (Table 4).

We also determined growth rates (i.e. production) of the most abundant individual zooplankton taxa across lake type in the mesocosm experiment. Calanoid production in non-manipulated lakes did not differ with DOC concentration, but was 24-fold lower in the control lakes in 2013 compared to 2011 (Fig. 6b; Table 4). N fertilization had an additional strong negative effect on calanoid production in the low DOC lakes, no effect on the medium DOC lake and a positive effect on the high DOC lake (Table 4). Cyclopoid production did not differ with DOC concentration in the control lakes (Fig. 6c). N fertilization resulted in a fivefold decrease in cyclopoid production (Table 4). However, cyclopoid growth was only reduced in low and medium DOC lakes and remained unchanged in the high DOC lake (Table 4). The growth rate of *Bosmina* sp. did not differ with DOC in non-manipulated lakes (Fig. 6d). In the control lakes, *Bosmina* growth decreased in 2013 in low and medium DOC lakes (47, and fourfold) and increased in the high DOC control lake (fivefold) (Table 4). N fertilization did not have any effect on *Bosmina* growth rate. *Ceriodaphnia* growth rate increased with increasing DOC, 15-fold from low to medium and 13-fold from medium to high DOC concentrations (Fig. 6e). Neither year, nor N fertilization influenced *Ceriodaphnia* growth.

The food web efficiency (FWE) differed between 2011 and 2013, but did not differ with DOC in non-manipulated lakes. FWE in control lakes in 2013 was sixfold lower than in 2011 (Fig. 4f; Table 4). N fertilization further decreased FWE and caused a ninefold decrease in FWE in the N-lakes (Table 4). This negative effect of N fertilization on FWE was strong in the low and medium DOC lakes, whereas a weak positive N effect was found in the high DOC lake (Table 4).

**Fig. 7:**
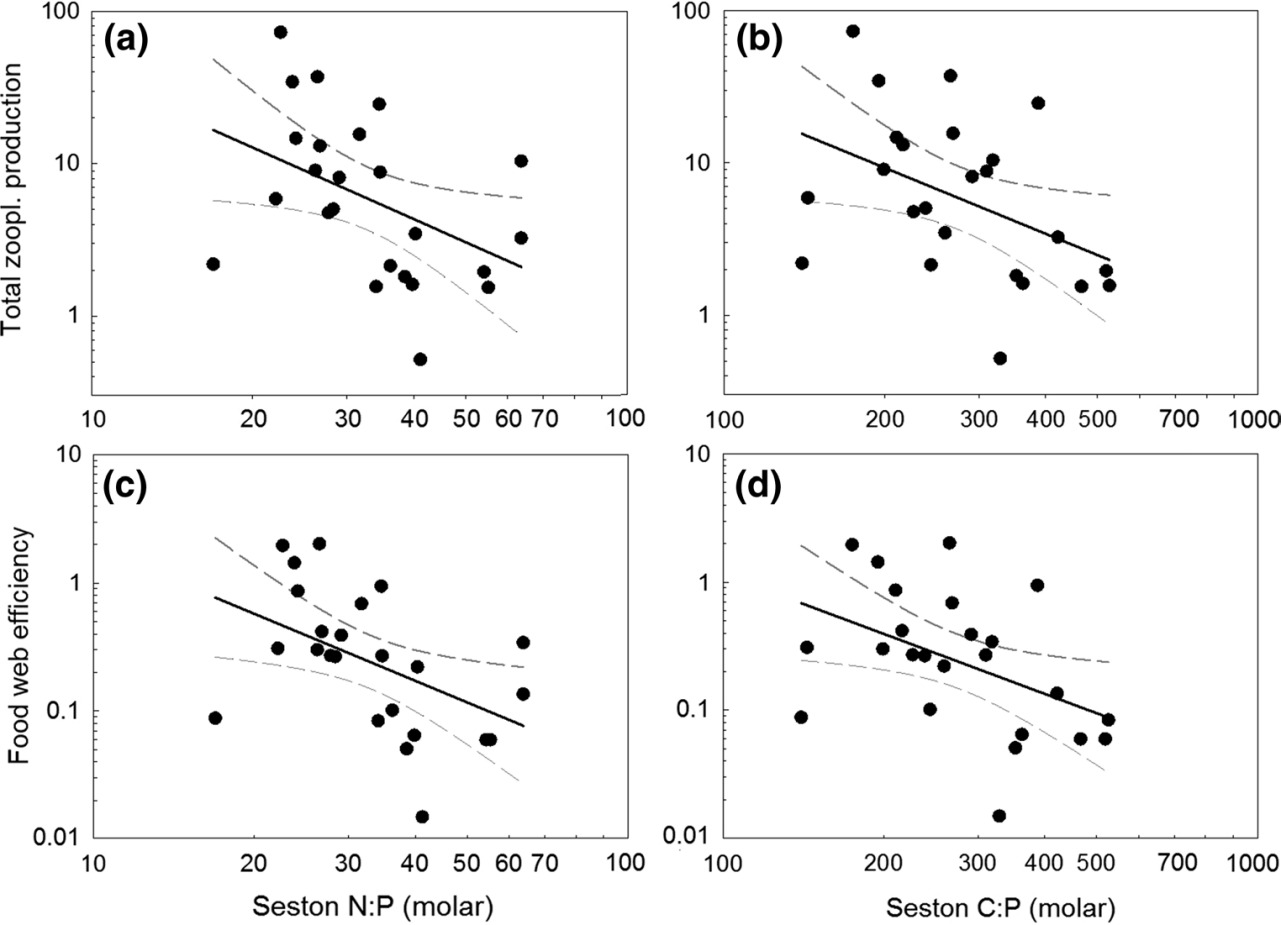
Linear regression between total zooplankton production (Tot. Zoopl. Production) and **a** seston N:P and **b** C:P, and between food web efficiency (FWE) and **c** seston N:P and **d** C:P during the time frame of the mesocosm experiment (July–August, *n* = 3) including 95% confidence intervals (*dashed*). Note log scale

Last, we found that both TZP and FWE decreased with increasing N:P and C:P ratios of seston (Fig. 7; Table 2).

## Discussion

Here, we show for the first time how increased N availability affects consumer growth and food web efficiency in N-limited unproductive boreal lakes. Our results illustrate that although primary production (PP) and pelagic energy mobilization (PEM) increased in all lakes following N fertilization (supporting hypothesis 1), the direction of the response of total zooplankton production (TZP) and food web efficiency (FWE) differed between lakes depending on the DOC concentration (rejecting hypothesis 2). In low and medium DOC lakes both TZP and FWE decreased with N fertilization, although phytoplankton production (PP) increased the most at these DOC levels and potentially resulted in a shorter, more efficient pelagic food chain as shown by increased PP:BP ratios (rejecting hypothesis 3). Instead, the decrease in crustacean zooplankton growth and FWE was associated with a decline in phytoplankton food quality at these DOC levels represented by higher seston C:P and N:P ratios, which seemed to have offset any positive effects of increased food quantity with fertilization. In contrast, in the high DOC lake, where the seston stoichiometry was comparably less affected by N fertilization, both TZP and FWE increased after N fertilization, despite only a modest increase in PP. Our results imply that the response of the pelagic food web to N fertilization will strongly depend on the lake-specific DOC concentration and its effects on seston stoichiometry.

In line with earlier studies our results show that phytoplankton in northern boreal lakes are N-limited and, therefore, responded positively to N fertilization (Jansson et al. 2001; Bergström et al. 2008; Elser et al. 2009a). As hypothesized, the net response of PP to N fertilization was lowest in the lake with highest DOC concentration. Although P availability increases with DOC (Table 1), light becomes increasingly limiting and thereby sets the threshold for net increases in PP following N fertilization (Faithfull et al. 2015; Seekell et al. 2015; Deininger et al. 2017a). Nevertheless, total pelagic energy mobilization (PEM) increased in all lakes after fertilization, but PEM did not decrease with increasing DOC concentrations as hypothesized. We attribute these differences to the stimulation of the microbial loop (Azam et al. 1983) fueling bacteria with algal exudates following enhanced PP caused by fertilization, wherefore BP increased in medium and high DOC lakes, but not in the low DOC lake, the latter likely due to lower P availability (Table 1) (Jones 1992). Consequently, N fertilization did not shorten the pelagic food web to the same extent in all lakes. Especially in the high DOC lake, the PP:BP ratio indicates that the pelagic system stayed net heterotrophic irrespective of N fertilization (i.e. PP:BP <1) (del Giorgio and Peters 1994). However, there was a pronounced shortening of the pelagic food chain with N fertilization in the low DOC lake since BP decreased and PP increased.

Despite N fertilization promoting high PP and a shorter food chain in the low and medium DOC lakes, TZP and FWE were reduced in these lakes. Only in the high DOC lake did the comparably modest increase in phytoplankton production result in both increased TZP and FWE. Based on the negative relationship between TZP and FWE and increasing seston C:P and N:P stoichiometry, we propose that the positive effects of increased food quantity and a shorter food chain length were offset by the negative impact of reduced food quality in response to N fertilization in the low and medium DOC lakes. Seston stoichiometry has been associated with consumer nutrient limitation, with threshold elemental ratios for nutrient limitation in cladocerans ranging between ca. 12–18 for N:P and 200–300 for C:P in *Daphnia* and >300 for C:P in *Bosmina* (Hessen and Lyche 1991; Urabe and Watanabe 1992; Sterner and Elser 2002). Similarly, N:P and C:P threshold elemental ratios in copepods have been estimated to be >30 and >300, respectively, due to their lower P requirements (Kibby 1971; Andersen and Hessen 1991). We observed a twofold increase in seston N:P ratio, and a C:P ratio >300 over the whole experimental period after N fertilization. This suggests that consumers grazing on phytoplankton became P limited in our experiment, especially in the low and medium DOC lakes where stoichiometric changes were largest (Figs. 3, 4) (Sterner et al. 1998; Elser et al. 2010; Hessen 2013). The observed changes in seston food quality are connected to increases in phytoplankton biomass in all lakes (cf. Figs. 4, 7a, b) (Deininger et al. 2017a) despite the lakes naturally differing in phytoplankton, bacterial and detritus contributions to seston carbon (Hessen et al. 2003). When calculating the contribution of phytoplankton biomass (Deininger et al. 2017a) to seston carbon, we found that N fertilization increased the proportion of phytoplankton carbon to seston carbon in all lakes (i.e. from 16 to 51% (low DOC), 25 to 45% (medium DOC) and 10 to 37% (high DOC)). Thus, although phytoplankton was a comparably minor contributor to seston carbon, especially in the high DOC lake, N fertilization clearly increased phytoplankton seston carbon relative to detritus even at this DOC level.

In support of our hypothesis that seston stoichiometry limited zooplankton production, we found that TZP and FWE showed the strongest decrease in the low DOC lake where the amount of poor quality food (i.e. seston C:P and N:P) increased the most following N fertilization (cf. Figs. 3, 4, 6). In contrast, TZP and FWE increased in the high DOC lake, despite the relatively small increases in phytoplankton and pelagic energy mobilization. A likely reason for the stable seston C:P ratio in the high DOC lake might be that lower light availability and thus light limitation prevented phytoplankton from building up seston C, despite high P availability (Deininger et al. 2017a) (cf. Table 2) and enabling higher P uptake, compared to the situation in the low DOC lake (i.e. high light availability and low P availability; Table 1) (Jones 1992; Sterner et al. 1997). Similarly, in the medium DOC lake, pelagic food web efficiency decreased much less with fertilization than in the low DOC lake.

Interestingly, zooplankton taxa responded differently to N fertilization along the lake DOC gradient, likely linked to their different feeding strategies and somatic N:P requirements. Responses to changes in food quantity and quality (Sterner et al. 1998; Persson et al. 2007; Elser et al. 2010) were especially apparent for selectively feeding calanoid copepods (Demott 1988; Tiselius and Jonsson 1990), which are typically specialized on phytoplankton prey during summer (Berggren et al. 2015). Further, previous analysis addressing zooplankton resource use in response to N fertilization showed that calanoid diet in these lakes is purely based on phytoplankton in summer (Deininger et al. 2017b). Indeed, growth of calanoids was strongly linked to the DOC-related changes in seston food quality. In the low DOC lake, where seston N:P and C:P ratios increased the most (>30 and >400, respectively) (cf. Figs. 3, 4), calanoid production showed the strongest decrease, implying P limitation in calanoids at this DOC level (Kibby 1971; Hessen and Lyche 1991). Likely, calanoid preadult life stages (nauplii, copepodites) were even more strongly affected, since their growth stages have higher P demands compared to adults and could have acted as a bottle neck for calanoid development (Villar-Argaiz and Sterner 2002; Bullejos et al. 2014a). The calanoid growth rates were related to changes in seston C:P and N:P ratios after fertilization, with growth rates showing only minor declines in medium DOC and even enhanced rates at high DOC concentrations in accordance with changes in C:P and N:P ratios in these lakes. Consequently, as high DOC lakes are darker and more nutrient rich, they appear to be more resilient to changes in phytoplankton stoichiometry following N enrichment. This is an interesting finding, since it suggests that although phytoplankton quantity increased only modestly and stoichiometric food quality did not change, calanoid diet was to some extent upgraded in the high DOC lake by N fertilization. Potentially, phytoplankton at this DOC level may have also provided calanoids with additional essential polyunsaturated fatty acids (PUFAs) (Brett and Müller-Navarra 1997; Müller-Navarra et al. 2000; Gladyshev et al. 2011), since lake DOC concentration influenced the phytoplankton community composition (with a dominance of non flagellated autotrophs at low DOC towards an increasing dominance of flagellated autotrophs with increased lake DOC), whereas phytoplankton community composition was unaffected by N fertilization (Deininger et al. 2017a). These differences in phytoplankton community composition and PUFA content with DOC may have influenced calanoid growth (Brett and Müller-Navarra 1997; Müller-Navarra et al. 2000; Gladyshev et al. 2011). Since calanoid copepods were the main zooplankton taxa contributing to the overall change in TZP after N fertilization (cf. Fig. 6a) our results imply that FWE in boreal lakes dominated by calanoids is sensitive to, and will be most affected by changes in inorganic N availability.

Cyclopoid copepods showed a similar trend to calanoids, and reduced growth was observed in both the low and medium DOC lake after N fertilization. Possibly cyclopoid growth was impaired even more by the decreased phytoplankton food quality since cyclopoids show higher P demands than calanoids (Andersen and Hessen 1991; Bullejos et al. 2014b). Also for this copepod taxa, earlier life stages might have been more severely affected by the induced P limitation resulting from N enrichment and acted as a possible bottleneck for cyclopoid development (Villar-Argaiz and Sterner 2002; Bullejos et al. 2014a).

Surprisingly, N fertilization did not affect the two dominant cladoceran species *Ceriodaphnia* and *Bosmina*, which were most abundant in medium and especially in the high DOC lakes. Theoretically, cladocerans should have been most sensitive to changes in phytoplankton food quality due to their higher P requirements compared to copepods (Andersen and Hessen 1991; Hessen and Lyche 1991). The unselective filter feeding mode of cladocerans prevents them from selecting high-quality phytoplankton food as efficiently as copepods (Sommer and Sommer 2006; Barnett et al. 2007). Therefore, cladoceran diet often reflects the relative availability of phytoplankton versus bacterial derived carbon (Karlsson et al. 2012; Deininger et al. 2017b). The most likely reason why cladoceran growth did not increase in the low DOC lake with N fertilization might be their low relative abundance at this DOC level (<20%), and we can thus not rule out that this led to an underestimation of the cladoceran response to N fertilization. Further studies are needed to investigate whether this missing response to the decreased phytoplankton food quality at low DOC is a universal response of cladocerans to increased N availability or merely an artifact related to the zooplankton community present in our experiment. At higher DOC levels, it is likely that feeding on P-rich bacteria (Andersen and Hessen 1991; Sterner and Elser 2002) enabled filter feeding cladoceran taxa to compensate for the induced P limitation caused by N fertilization since bacterial production and TP availability (cf. Table 1; Fig. 2) were higher at these DOC levels.

In summary, our findings suggest that phytoplankton food quality has a strong effect on zooplankton fitness, especially for taxa that are primarily phytoplankton grazers. Further, DOC, by influencing light and nutrient availability, is a strong indirect force modifying the response of zooplankton consumers to increased N availability by affecting phytoplankton C:N:P stoichiometry.

The decline in zooplankton growth and FWE (estimated via the mesocosm experiment), but also zooplankton biomass (estimated outside the mesocosms) in 2013 compared to 2011, suggests that seasonality was a strong driver on the zooplankton community. Large seasonal and yearly variations in zooplankton population dynamics have been reported earlier and both abiotic (e.g. temperature) and biotic factors (e.g. predation, food availability, and quality) have been discussed as potential causes (Hessen et al. 1995; Elser et al. 1998; Jansson et al. 2001). Nevertheless, due to the before–after control-impact design of our experiment we were able to separate between seasonal/yearly effects and fertilization effects (Carpenter et al. 1989), even though seasonal variability was high between the experimental periods. However, we can only speculate about the response of zooplankton consumers and FWE to conditions similar to the reference year 2011. Potentially, given the same abiotic conditions in 2013 and 2011, zooplankton response to N fertilization might have been even larger, due to the potentially stronger responses of phytoplankton given similarly high light levels as in 2011.

In conclusion, our results clearly illustrate that in the pelagic zone of unproductive boreal lakes the effects of increased N availability on FWE and zooplankton production will differ depending on background lake DOC concentrations. The positive effects of increased N availability such as increased food quantity and shortening of the pelagic food chain caused by elevated PP will be outweighed by the negative effects of increased availability of poor quality phytoplankton with increased N:P and C:P stoichiometry. As a consequence, zooplankton growth and FWE will be impaired. However, with increasing DOC concentrations, the negative effects of N fertilization on phytoplankton stoichiometry will become smaller, since nutrient concentrations will increase and light availability will decrease. Thus, terrestrial derived DOC will determine the net effects of increased inorganic N on the energy transfer of boreal lake food webs: first, by influencing light availability and thus, phytoplankton production, community composition (Deininger et al. 2017a) and stoichiometry, and second by providing an external energy source for bacteria and enhancing P availability. Last, the zooplankton community composition might play an important role in determining the overall response of consumers to enhanced N availability. Especially communities dominated by herbivorous species such as calanoids will be sensitive to changes in phytoplankton food quality. In summary, our study provides the first important insights as to how unproductive boreal lake food webs will respond to increases in N availability, further taking variations in DOC concentrations into account. Our results suggest that clear and humic lakes function very differently: increased N availability will decrease energy transfer in clear lakes caused by mismatches in food quality demand and supply. In humic lakes this mismatch will not occur, wherefore zooplankton production and FWE will increase following enhanced N availability.

## References

[CR1] Andersen T, Hessen DO (1991). Carbon, nitrogen, and phosphorus-content of fresh-water zooplankton. Limnol Oceanogr.

[CR2] Azam F, Fenchel T, Field JG, Gray JS, Meyerreil LA, Thingstad F (1983). The ecological role of water-column microbes in the sea. Mar Ecol Prog Ser.

[CR3] Barnett AJ, Finlay K, Beisner BE (2007). Functional diversity of crustacean zooplankton communities: towards a trait-based classification. Freshw Biol.

[CR4] Berggren M, Bergström A-K, Karlsson J (2015). Intraspecific autochthonous and allochthonous resource use by zooplankton in a Humic Lake during the transitions between winter, summer and fall. PLoS One.

[CR5] Berglund J, Muren U, Båmstedt U, Andersson A (2007). Efficiency of a phytoplankton-based and a bacteria-based food web in a pelagic marine system. Limnol Oceanogr.

[CR6] Bergström AK, Blomqvist P, Jansson M (2005). Effects of atmospheric nitrogen deposition on nutrient limitation and phytoplankton biomass in unproductive Swedish lakes. Limnol Oceanogr.

[CR7] Bergström AK, Jonsson A, Jansson M (2008). Phytoplankton responses to nitrogen and phosphorus enrichment in unproductive Swedish lakes along a gradient of atmospheric nitrogen deposition. Aquat Biol.

[CR8] Bergström AK, Faithfull C, Karlsson D, Karlsson J (2013). Nitrogen deposition and warming—effects on phytoplankton nutrient limitation in subarctic lakes. Glob Change Biol.

[CR9] Bergström A-K, Karlsson D, Karlsson J, Vrede T (2015). N-limited consumer growth and low nutrient regeneration N:P ratios in lakes with low N deposition. Ecosphere.

[CR10] Bottrell HH, Duncan A, Gliwicz ZM, Grygierek E, Herzig A, Hillbrichtilkowska A, Kurasawa H, Larsson P, Weglenska T (1976). Review of some problems in zooplankton production studies. Nor J Zool.

[CR11] Brett MT, Müller-Navarra DC (1997). The role of highly unsaturated fatty acids in aquatic food web processes. Freshw Biol.

[CR12] Bullejos FJ, Carrillo P, Gorokhova E, Medina-Sanchez JM, Balseiro EG, Villar-Argaiz M (2014). Shifts in food quality for herbivorous consumer growth: multiple golden means in the life history. Ecology.

[CR13] Bullejos FJ, Carrillo P, Gorokhova E, Medina-Sanchez JM, Villar-Argaiz M (2014). Nucleic acid content in Crustacean zooplankton: bridging metabolic and stoichiometric predictions. PLoS One.

[CR14] Carpenter SR, Frost TM, Heisey D, Kratz TK (1989). Randomized intervention analysis and the interpretation of whole-ecosystem experiments. Ecology.

[CR15] de Wit HA, Valinia S, Weyhenmeyer GA, Futter MN, Kortelainen P, Austnes K, Hessen DO, Räike A, Laudon H, Vuorenmaa J (2016). Current browning of surface waters will be further promoted by wetter climate. Environ Sci Technol Lett.

[CR16] Deininger A, Faithfull CL, Bergström AK (2017). Phytoplankton response to whole lake inorganic N fertilization along a gradient in dissolved organic carbon. Ecology.

[CR17] Deininger A, Faithfull CL, Karlsson J, Klaus M, Bergström AK (2017). Pelagic food web response to whole lake N fertilization. Limnol Oceanogr.

[CR18] del Giorgio PA, Peters RH (1994). Patterns in planktonic P-R ratios in lakes—influence of lake trophy and dissolved organic-carbon. Limnol Oceanogr.

[CR19] Demott WR (1988). Discrimination between algae and artifical particles by fresh-water and marine copepods. Limnol Oceanogr.

[CR20] Dickman EM, Newell JM, Gonzalez MJ, Vanni MJ (2008). Light, nutrients, and food-chain length constrain planktonic energy transfer efficiency across multiple trophic levels. Proc Natl Acad Sci USA.

[CR21] Downing JA, Prairie YT, Cole JJ, Duarte CM, Tranvik LJ, Striegl RG, McDowell WH, Kortelainen P, Caraco NF, Melack JM, Middelburg JJ (2006). The global abundance and size distribution of lakes, ponds, and impoundments. Limnol Oceanogr.

[CR22] Elser JJ, Chrzanowski TH, Sterner RW, Mills KH (1998). Stoichiometric constraints on food-web dynamics: a whole-lake experiment on the Canadian shield. Ecosystems.

[CR23] Elser JJ, Andersen T, Baron JS, Bergström AK, Jansson M, Kyle M, Nydick KR, Steger L, Hessen DO (2009). Shifts in lake N: P stoichiometry and nutrient limitation driven by atmospheric nitrogen deposition. Science.

[CR24] Elser JJ, Kyle M, Steger L, Nydick KR, Baron JS (2009). Nutrient availability and phytoplankton nutrient limitation across a gradient of atmospheric nitrogen deposition. Ecology.

[CR25] Elser JJ, Peace AL, Kyle M, Wojewodzic M, McCrackin ML, Andersen T, Hessen DO (2010). Atmospheric nitrogen deposition is associated with elevated phosphorus limitation of lake zooplankton. Ecol Lett.

[CR26] Faithfull C, Huss M, Vrede T, Karlsson J, Bergström AK (2012). Transfer of bacterial production based on labile carbon to higher trophic levels in an oligotrophic pelagic system. Can J Fish Aquat Sci.

[CR27] Faithfull CL, Mathisen P, Wenzel A, Bergström AK, Vrede T (2015). Food web efficiency differs between humic and clear water lake communities in response to nutrients and light. Oecologia.

[CR28] Finstad AG, Andersen T, Larsen S, Tominaga K, Blumentrath S, de Wit HA, Tømmervik H, Hessen DO (2016). From greening to browning: catchment vegetation development and reduced S-deposition promote organic carbon load on decadal time scales in Nordic lakes. Sci Rep.

[CR29] Gladyshev MI, Sushchik NN, Anishchenko OV, Makhutova ON, Kolmakov VI, Kalachova GS, Kolmakova AA, Dubovskaya OP (2011). Efficiency of transfer of essential polyunsaturated fatty acids versus organic carbon from producers to consumers in a eutrophic reservoir. Oecologia.

[CR30] Greaver TL, Clark CM, Compton JE, Vallano D, Talhelm AF, Weaver CP, Band LE, Baron JS, Davidson EA, Tague CL, Felker-Quinn E, Lynch JA, Herrick JD, Liu L, Goodale CL, Novak KJ, Haeuber RA (2016). Key ecological responses to nitrogen are altered by climate change. Nat Clim Change.

[CR31] Hessen DO (2013). Inorganic nitrogen deposition and its impacts on N:P-ratios and lake productivity. Water.

[CR32] Hessen DO, Lyche A (1991). Interspecific and intraspecific variations in zooplankton element composition. Arch Hydrobiol.

[CR33] Hessen DO, Faafeng BA, Andersen T (1995). Replacement of herbivore zooplankton species along gradients of ecosystem productivity and fish predation pressure. Can J Fish Aquat Sci.

[CR34] Hessen DO, Andersen T, Brettum P, Faafeng BA (2003). Phytoplankton contribution to sestonic mass and elemental ratios in lakes: implications for zooplankton nutrition. Limnol Oceanogr.

[CR35] Jansson M, Bergström AK, Blomqvist P, Drakare S (2000) Allochthonous organic carbon and phytoplankton/bacterioplankton production relationships in lakes. Ecology 81:3250–3255. doi:10.1890/0012-9658(2000)081[3250:aocapb]2.0.co;2

[CR36] Jansson M, Bergström AK, Drakare S, Blomqvist P (2001). Nutrient limitation of bacterioplankton and phytoplankton in humic lakes in northern Sweden. Freshw Biol.

[CR37] Jansson M, Persson L, De Roos AM, Jones RI, Tranvik LJ (2007). Terrestrial carbon and intraspecific size-variation shape lake ecosystems. Trends Ecol Evol.

[CR38] Jones RI (1992). The influence of humic substances on lacustrine planktonic food-chains. Hydrobiologia.

[CR39] Jones SE, Solomon CT, Weidel BC (2012). Subsidy or subtraction: how do terrestrial inputs influence consumer production in lakes?. Freshw Rev.

[CR40] Karlsson J, Jansson M, Jonsson A (2002). Similar relationships between pelagic primary and bacterial production in clearwater and humic lakes. Ecology.

[CR41] Karlsson J, Berggren M, Ask J, Bystrom P, Jonsson A, Laudon H, Jansson M (2012). Terrestrial organic matter support of lake food webs: evidence from lake metabolism and stable hydrogen isotopes of consumers. Limnol Oceanogr.

[CR42] Kibby HV (1971). Energetics and population dynamics of *Diaptomus*-gracilis. Ecol Monogr.

[CR43] Kreutzweiser DP, Hazlett PW, Gunn JM (2008). Logging impacts on the biogeochemistry of boreal forest soils and nutrient export to aquatic systems: a review. Environ Rev.

[CR44] Kritzberg ES, Cole JJ, Pace MM, Graneli W (2006). Bacterial growth on allochthonous carbon in humic and nutrient-enriched lakes: results from whole-lake C-13 addition experiments. Ecosystems.

[CR45] Mason CF, Abdulhussein MM (1991). Population-dynamics and production of *Daphnia-hyalina* and *Bosmina-longirostris* in a shallow, eutrophic reservoir. Freshw Biol.

[CR46] Moldan F, Kjonaas OJ, Stuanes AO, Wright RF (2006). Increased nitrogen in runoff and soil following 13 years of experimentally increased nitrogen deposition to a coniferous-forested catchment at Gardsjon, Sweden. Environ Pollut.

[CR47] Monteith DT, Stoddard JL, Evans CD, de Wit HA, Forsius M, Hogasen T, Wilander A, Skjelkvale BL, Jeffries DS, Vuorenmaa J, Keller B, Kopacek J, Vesely J (2007). Dissolved organic carbon trends resulting from changes in atmospheric deposition chemistry. Nature.

[CR48] Müller-Navarra DC, Brett MT, Liston AM, Goldman CR (2000). A highly unsaturated fatty acid predicts carbon transfer between primary producers and consumers. Nature.

[CR49] Oksanen J, Blanchet FG, Kindt R, Legendre P, Minchin PR, O’Hara RB, Simpson GL, Solymos P, Stevens MHH, Wagner H (2016) Vegan: Community Ecology Package. R., vol. R package version 2.3-3

[CR50] Persson J, Brett MT, Vrede T, Ravet JL (2007). Food quantity and quality regulation of trophic transfer between primary producers and a keystone grazer (*Daphnia*) in pelagic freshwater food webs. Oikos.

[CR51] Pinheiro J, Bates D, DebRoy S, Sarkar D (2009) Nlme: linear and nonlinear mixed effects models. R., vol. Package Version 3, 1–96. package=nlme

[CR52] Rockström J, Steffen W, Noone K, Persson A, Chapin FS, Lambin EF, Lenton TM, Scheffer M, Folke C, Schellnhuber HJ, Nykvist B, de Wit CA, Hughes T, van der Leeuw S, Rodhe H, Sorlin S, Snyder PK, Costanza R, Svedin U, Falkenmark M, Karlberg L, Corell RW, Fabry VJ, Hansen J, Walker B, Liverman D, Richardson K, Crutzen P, Foley JA (2009). A safe operating space for humanity. Nature.

[CR53] Schindler DW, Schmidt RV, Reid RA (1972). Acidification and bubbling as an alternative to filtration in determining phytoplankton production by C-14 method. J Fish Res Board Can.

[CR54] Seekell DA, Lapierre J-F, Ask J, Bergström A-K, Deininger A, Rodriguez P, Karlsson J (2015). The influence of dissolved organic carbon on primary production in northern lakes. Limnol Oceanogr.

[CR55] SMHI (2016) Kartläggning lufthalt och deposition. (’Mapping of atmospheric concentrations and deposition’). Swedish Meteorological and Hydrological Institute. Available at: http://www.smhi.se/klimatdata/miljo/atmosfarskemi. Accessed 29 Aug 2016

[CR56] Smith DC, Azam F (1992). A simple, economical method for measuring bacterial protein synthesis rates in seawater using 3H-leucine. Mar Microb Food Webs.

[CR57] Sobek S, Tranvik LJ, Prairie YT, Kortelainen P, Cole JJ (2007). Patterns and regulation of dissolved organic carbon: an analysis of 7,500 widely distributed lakes. Limnol Oceanogr.

[CR58] Solomon CT, Jones SE, Weidel BC, Buffam I, Fork ML, Karlsson J, Larsen S, Lennon JT, Read JS, Sadro S, Saros JE (2015). Ecosystem consequences of changing inputs of terrestrial dissolved organic matter to lakes: current knowledge and future challenges. Ecosystems.

[CR59] Sommer U, Sommer F (2006). Cladocerans versus copepods: the cause of contrasting top-down controls on freshwater and marine phytoplankton. Oecologia.

[CR60] Sterner RW, Elser JJ (2002). Ecological Stoichiometry: the biology of elements from molecules to the biosphere.

[CR61] Sterner RW, Elser JJ, Fee EJ, Guildford SJ, Chrzanowski TH (1997). The light:nutrient ratio in lakes: the balance of energy and materials affects ecosystem structure and process. Am Nat.

[CR62] Sterner RW, Clasen J, Lampert W, Weisse T (1998). Carbon: phosphorus stoichiometry and food chain production. Ecol Lett.

[CR63] Tiselius P, Jonsson PR (1990). Foraging behavior of 6 calanoid copepods—observations and hydrodynamic analysis. Mar Ecol Prog Ser.

[CR64] Tranvik LJ (1988). Availability of dissolved organic-carbon for planktonic bacteria in oligotrophic lakes of differing humic content. Microb Ecol.

[CR65] Urabe J, Watanabe Y (1992). Possibility of N-limitation or P-limitation for planktonic Cladocerans—an experimental test. Limnol Oceanogr.

[CR66] Villar-Argaiz M, Sterner RW (2002). Life history bottlenecks in *Diaptomus* clavipes induced by phosphorus-limited algae. Limnol Oceanogr.

[CR67] Vitousek PM, Mooney HA, Lubchenco J, Melillo JM (1997). Human domination of Earth’s ecosystems. Science.

[CR68] Wetzel RG (2001). Plankton communities: Algae and Cyanobacteria. Limnology. Lake and river ecosystems.

